# Sarcotubular Myopathy Due to Novel *TRIM32* Mutation in Association with Multiple Sclerosis

**DOI:** 10.3390/brainsci11081020

**Published:** 2021-07-31

**Authors:** Margarita Marchuk, Tetiana Dovbonos, Halyna Makukh, Orest Semeryak, Yevheniya Sharhorodska

**Affiliations:** 1 Department of Neurology №2, Kyiv City Clinical Hospital №4, 03110 Kiev, Ukraine; 2 Department of Neurology, O.O. Bogomolets National Medical University, 13 Shevchenko Blvd., 01601 Kyiv, Ukraine; 3 Institute of Hereditary Pathology of National Academy of Medical Sciences of Ukraine, 31A Lysenka Str., 79008 Lviv, Ukraine; makukh_halyna@ukr.net; 4 Center for Neuromuscular and Peripheral Nervous System Diseases, Lviv Regional Clinical Hospital, 7 Chernihivska Str., 79010 Lviv, Ukraine; dr.orchyk@gmail.com; 5 Institute of Hereditary Pathology of National Academy of Medical Sciences of Ukraine, 31A Lysenka Str., 79008 Lviv, Ukraine; gendoctor86@gmail.com

**Keywords:** LGMD2H, LGMD R8, Limb-Girdle Muscular Dystrophy 2H, sarcotubular myopathy, TRIM32, muscular dystrophy, multiple sclerosis, Hoover’s rising, waddling gait, next-generation sequencing

## Abstract

Azerbaijani 28-year-old female showed weakness (MRC (Medical Research Council Scale for Muscle Strength) grade 4 in the proximal part of the upper and MRC grade 2–3 in the lower extremities), difficulty in stair lifting, positive symptom of Hoover’s rising, «waddling gait», decline deep reflexes symmetrical, lack of surface reflexes, positive Babinsky’s reflex on the right, urinary incontinence during sneezing, prolonged walking and exercise from puberty. Additional methods made it possible to identify minor violations of conduction of the left ventricle, electromyography signs of primary muscular disease with predominant involvement of the proximal muscles of the lower extremities, elevation of serum creatine kinase (746.81 U/l), active foci of demyelination in the left frontal lobe, intrathecal synthesis of oligoclonal IgG bands (type 2) in cerebrospinal fluid, atrophy and fatty degeneration of all muscles of the shins, homozygous Variant of Uncertain Significance (VUS) c.1855C > T (p.Pro619Ser) in *TRIM32* gene and heterozygous VUS c.2300C > G (p.Thr767Arg) in *KIF5A*, c.2840G > A (p.Arg947Lys) in *MYH2*, c.1502G > C (p.Gly501Ala) in *POMT1* genes. Comparison of the phenotypes of the mutations that have been identified with the clinical picture of the patient suggests that VUS c.1855C > T (p.Pro619Ser) in the *TRIM32* gene can be pathological. Summarizing, it can be argued that the cause of the identified disorders is a homozygous variant c.1855C > T (p.Pro619Ser) in *TRIM32* gene that causes LGMDR8 in a patient with MS.

## 1. Introduction

*Tripartite motif-containing protein 32 (TRIM32)* is a member of the TRIM ubiquitin E3 ligases which ubiquitinates different substrates in muscle including sarcomeric proteins. Mutations in *TRIM32* have been associated to Limb-Girdle Muscular Dystrophy 2H (LGMD2H) and Sarcotubular Myopathy, a form of distal myopathy with peculiar features in muscle biopsy, now considered in the spectrum of LGMD2H. [1]. TRIM32 mutations were initially described in the Manitoba Hutterite population (41 patients) of North America presenting with a phenotype of the LGMD2H and the first mutation identified was the c.1459G > A (p. Asp487Asn) [2]. However, in 2018 after the overall development of molecular-genetic research, classification of LGMD became reorganized resulting in renaming LGMD2H to Limb-Girdle Muscular Dystrophy, Autosomal Recessive 8 (LGMDR8) [3]. For today, LGMDR8 is a rare muscular dystrophy due to mutation in *TRIM32* gene with a wide clinical presentation spectrum [2,4,5,6]. Additionally, the problem of comorbidity is not covered in the literature, especially for multiple sclerosis (MS) and LGMDR8. 

## 2. Case Report

The collection of complaints of Azerbaijani 28-year-old female showed weakness and fatigue of limb muscles, difficulty in stair climbing, prolonged walking and exercise. For the first time in physical education classes at school, she noticed that she could not fulfill standards that were simple for others. Since puberty, weakness in the lower extremities has progressed. The patient was limited in the ability to walk for a long time, gradually there was a significant difficulty when climbing stairs, getting up from a chair, then finally she could not rise from a squatting position without the support on the objects around her or the help of others. 

During the neurological examination there was detected decrease in strength mainly in the proximal part of the upper (Medical Research Council Scale for Muscle Strength - MRC grade 4) and to a greater extent in the lower extremities (MRC grade 2–3), positive symptom of Hoover’s rising, «waddling gait», minor hypotrophy of the proximal limb muscles, decline of deep reflexes symmetrical.

**Figure 1:**
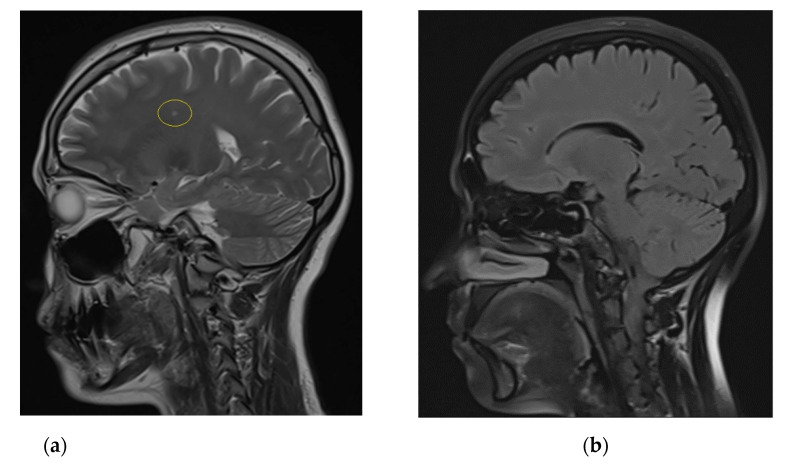
(**a**) Sagittal T2-weighted MRI scan of the brain shows active foci of demyelination in the left frontal lobe (yellow circle); (**b**) Sagittal T2 TIRM dark fluid MRI scan shows moderate tongue muscle atrophy.

Additionally, the neurologist’s attention was drawn to the small-amplitude nystagmus, lack of surface reflexes, positive Babinsky’s reflex on the right, urinary incontinence during sneezing. The discovered symptoms prompted specialists to conduct magnetic resonance imaging (MRI) of the brain. There were revealed active foci of demyelination in the left frontal lobe (Figure 1а). Re-examination after 2 months revealed multiple (>10) rounded and elongated foci hyperintensive on T2-weighted imaging (T2WI), fluid-attenuated inversion recovery (FLAIR) and short TI inversion recovery, iso- and hypointensive on T1-weighted imaging (T1WI), with a diameter of 2.5 mm to 5.8 mm, located periventricularly and subcortically in the white matter of the left temporal lobe and parietal lobes, frontal lobes, mostly in the left frontal lobe, as well as a decrease in the size of a previously active lesion and the appearance of new lesions in the right frontal and parietal regions. The isoelectric focusing detected an intrathecal synthesis of oligoclonal IgG bands (type 2) in cerebrospinal fluid. The results obtained confirm the diagnosis of relapsing-remitting MS.

However, the etiology of progressive symmetrical girdle weakness of the upper and lower extremities remained unknown. 

Electromyography (EMG) revealed the signs of primary muscular disease with predominant involvement of the proximal muscles of the lower extremities. 

Electrocardiography revealed minor violations of myocardial conduction of the left ventricle. 

Biochemical analysis showed elevation of serum creatine kinase (746.81 U/l). 

**Figure 2:**
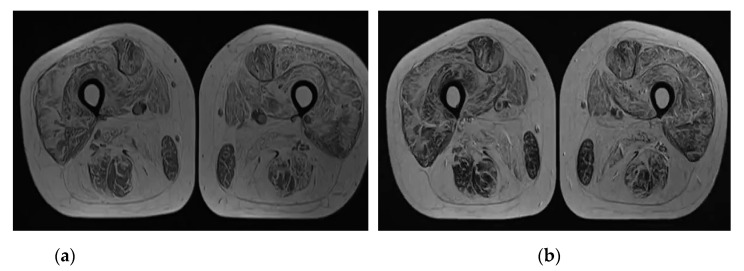
(**a**) Axial T1-weighted MRI scan with diffuse increase of signal intensity from all muscles of the both shins; (**b**) Axial T2 weighted MRI scan with diffuse increase of signal intensity from all muscles of the both shins.

**Figure 3:**
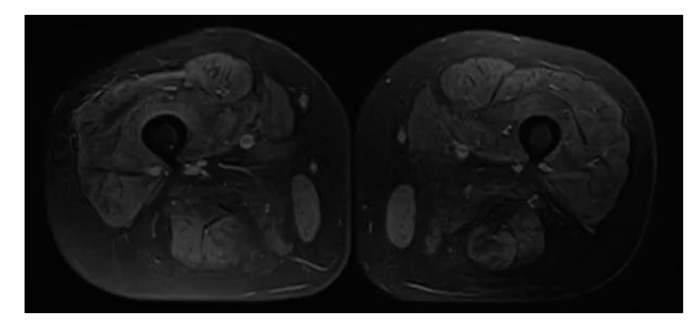
Axial fat-saturated proton density-weighted MRI scan with diffuse decrease of signal intensity from all muscles of the both shins.

MRI of the both shins revealed signs of muscular atrophy and fatty degeneration (Figure 2 and Figure 3) and MRI of the head shows moderate tongue muscle atrophy (Figure 1b). The MRI signal from other soft tissues is not changed. 

Tandem mass spectrometry from dried blood spot found a normal amount of alpha-1,4 glucosidase activity which made it possible to exclude the Pompe disease.

Next-generation sequencing (NGS) was chosen for more accurate diagnostics. Genomic DNA obtained from the submitted sample was enriched for targeted regions using a hybridization-based protocol, and sequenced using Illumina technology. Sequence analysis and deletion/duplication testing of the 305 genes Invitae Comprehensive Neuromuscular Disorders Panel was implemented. All targeted regions were sequenced with ≥50× depth or supplemented with additional analysis. All clinically significant observations were confirmed by orthogonal technologies. Confirmation technologies included Sanger sequencing, Multiplex ligation-dependent probe amplification. Technical component of confirmatory sequencing was performed by Invitae Corporation (San Francisco, CA, USA, 94103, #05D2040778).

NGS showed homozygous Variant of Uncertain Significance (VUS) c.1855C > T (p.Pro619Ser) in *TRIM32* gene and heterozygous VUS c.2300C > G (p.Thr767Arg) in *KIF5A*, c.2840G > A (p.Arg947Lys) in *MYH2*, c.1502G > C (p.Gly501Ala) in *POMT1* genes (Table 1).

Variant c.1855C > T (p.Pro619Ser) is located in exon 2 of *TRIM32* gene and caused the replacement of proline with serine at codon 619 of the TRIM32 protein (p.Pro619Ser). The proline residue is highly conserved and there is a moderate physicochemical difference between proline and serine. This variant is not present in population databases and it has not been reported in the literature in individuals with *TRIM32*-related conditions.

Variant c.2300C > G (p.Thr767Arg) of *KIF5A* gene causes the replacement of threonine with arginin at codon767 of the KIF5A protein (p.Thr767Arg). This variant is present in population databases (rs765493045, ExAC 0.006%).

The comparison of the phenotypes presented in Table 2 with the clinical picture of the patient suggests that VUS c.1855C > T (p.Pro619Ser) in the *TRIM32* gene can be pathological.

Additionally, according to the Human Phenotype Ontology (HPO) *Trim32*-related LGMDR8 is a mild subtype of autosomal recessive limb girdle muscular dystrophy characterized by slowly progressive proximal muscle weakness and wasting of the pelvic and shoulder girdles with onset that usually occurs during the second or third decade of life. Clinical presentation is variable and presented in Table 3. Other symptoms according to the HPO could be calf psuedohypertrophy, joint contractures, scapular winging, muscle cramping and/or facial and respiratory muscle involvement [24].

Summarizing, it can be argued that the cause of the identified disorders is a homozygous variant c.1855C > T (p.Pro619Ser) in *TRIM32* gene that causes LGMDR8 in a patient with MS.

Insofar as the systematic review demonstrated that effects on the clinical course and the long-term safety of gene transfer, myoblast transplantation, neutralizing antibody to myostatin, or growth hormone are yet to be determined the best supportive strategy includes:

- performing pulmonary function testing, detection of excessive daytime somnolence, nonrestorative sleep, episodes of syncope, near-syncope, palpitations, symptomatic or asymptomatic tachycardia or arrhythmias, or signs and symptoms of cardiac failure for cardiology evaluation (Level B).
- realization of periodic assessments by a physical and occupational therapist for symptomatic and preventive screening (Level B).
- combination of aerobic exercise with a supervised submaximal strength (Level C).
- practicing gentle, low-impact aerobic exercise (swimming, stationary bicycling) to improve cardiovascular performance, increase muscle efficiency, and lessen fatigue (Level C).
- adequate hydration, no exercise to exhaustion, and avoiding supramaximal, high-intensity exercise (Level C).
- detection of warning signs of overwork weakness and myoglobinuria, which include feeling weaker rather than stronger within 30 minutes after exercise, excessive muscle soreness 24–48 hours following exercise, severe muscle cramping, heaviness in the extremities, and prolonged shortness of breath (Level B) [25].

Expectedly, the weakness of pelvic girdle and shoulder muscles is in a state of slow progression. However, despite the worsening, she successfully graduated from university, passed the gestation period of her second child and had a cesarean section. The patient walks on her own, does not require assistance when climbing a short set of stairs, takes care of herself, and helps family members. She is regularly monitored by doctors, undergoes preventive examinations, is engaged in physical exercises and periodically receives specific treatment for MS.

## 3. Discussion

We describe a novel mutation in *TRIM32* gene in an adult patient who was presented with the combination of MS and a moderate limb muscles weakness which was regarded as LGMDR8.

The diagnosis of MS is confirmed by the 2017 McDonald criteria, according to which the presence of 1 attack and clinical evidence of 2 or more lesions in combination with new T2 or enhancing MRI lesion compared to baseline scan (without regard to timing of baseline scan) or oligoclonal bands in cerebrospinal fluid is the basis for the diagnosis [26]. With regard to limb muscle weakness in MS, systematic review and meta-analysis of cross-sectional studies confirmed the presence of neurophysiological decrements, manifest only as impaired maximum voluntary contraction force, reduced skeletal muscle voluntary activation and greater motor fatigability [27]. However, in our case, neurophysiological examination revealed a “myopathic type” of motor unit recruitment, which is EMG-characteristic of primary muscle disease.

The NGS analysis revealed in the patient the VUS in four genes: c.2300C > G (p.Thr767Arg) in *KIF5A*, c.2840G > A (p.Arg947Lys) in *MYH2*, c.1502G > C (p.Gly501Ala) in *POMT1* and c.1855C > T (p.Pro619Ser) in *TRIM32*. None of these variants were reported in patients with neuromuscular disorders. 

Compare to all heterozygous VUS which were discovered only c.1855C > T (p.Pro619Ser) in *TRIM32* is homozygous. As known the *POMT1* gene is associated with autosomal recessive disorder. The pathogenic variants in *KIF5A* and *MYH2* genes are associated with autosomal dominant conditions but not one (hereditary spastic paraplegia or body myopathy type 3) is clinically consistent with symptoms of the patient. Moreover, the replacement of arginine with lysine at codon 947 of the *MYH2* protein (p.Arg947Lys) leads to small physicochemical difference between amino acids.

The homozygous c.1855C > T (p.Pro619Ser) variant in *TRIM32* gene supports the hypothesis of autosomal recessive conditions. There were no cases of neuromuscular conditions among patients’ relatives. The patient is of Azerbaijani ethnicity and both of her parents have the same origin. It is important to emphasize that Azerbaijani population has high level of inbreeding marriage, which may cause the high risk of homozygosity for pathogenic variants [28]. We hypothesize that both parents of the patient have the same ancestors with c.1855C > T (p.Pro619Ser) variant in *TRIM32* gene. Unfortunately, the patient’s relatives refused to undergo examination due to the absence of symptoms. Genetic counseling did not discourage the relatives of the importance of testing. Therefore, the investigation of the pathogenic potential of the identified variants can be promising in the process of further monitoring of the patient. The variant is not described in clinical database and was not reported in the literature. LGMDR8 has autosomal recessive inheritance and cause by homozygous or compound heterozygous mutations in *TRIM32* gene. In the case we describe homozygous mutation in patient with clinical phenotype of LGMDR8. 

Our data support the reclassification of homozygous variant c.1855C > T (p.Pro619Ser) in *TRIM32* gene as pathogenic.

## Figures and Tables

**Table 1:** Identified variants of uncertain significance.

Gene	Variant	Zygosity	Variant Classification
*KIF5A*	c.2300C > G (p.Thr767Arg)	heterozygous	Uncertain Significance
*MYH2*	c.2840G > A (p.Arg947Lys)	heterozygous	Uncertain Significance
*POMT1*	c.1502G > C (p.Gly501Ala)	heterozygous	Uncertain Significance
*TRIM32*	c.1855C > T (p.Pro619Ser)	homozygous	Uncertain Significance

**Table 2:** Phenotypes of the detected mutations.

Gene	Diagnosis	Phenotype
*KIF5A* (Kinesin heavy chain isoform 5A)	Autosomal dominant hereditary spastic paraplegia 10 (SPG10) (MedGen UID: 349003)	’Pure’ spastic paraplegia: lower limb spasticity, hyperreflexia, extensor plantar responses variable involvement of the upper limbs appear in childhood or young adulthood axonal sensorimotor peripheral neuropathy: distal sensory impairment, muscle atrophy is reminiscent of Charcot-Marie-Tooth disease type 2 parkinsonism or cognitive decline [7,8]
	Intractable neonatal myoclonus (MedGen UID: 934625)	Intractable myoclonic seizures intermittent apnea abnormal eye movements pallor of the optic nerve lack of developmental progress appear after birth progressive leukoencephalopathy (brain imaging) death in infancy [9]
	Amyotrophic lateralsclerosis 25 (ALS25) (MedGen UID: 1534540)	Adult onset of focal asymmetric involvement of upper and lower motor neuron systems with later generalization bulbar motor involvement rapidly progressive muscle weakness death due to respiratory failure [10]
*MYH2* (Myosin-2)	Autosomal dominant and recessive inclusion body myopathy type 3 (MYPOP) (MedGen UID: 381340)	Proximal myopathy ophthalmoplegia childhood onset of symptoms slowly progressive or nonprogressive [11,[12]]
*POMT1*(Protein O-mannosyl-transferase 1)	Autosomal recessive muscular dystrophy-dystroglycanopathy type A1 (MDDGA1) (MedGen UID: 75553)	Brain and eye malformations: mental retardation, congenital muscular dystrophy, and early death cobblestone (type II) lissencephaly, cerebellar malformations, and retinal malformations macrocephaly or microcephaly, hypoplasia of midline brain structures, ventricular dilatation, microphthalmia, cleft lip/palate, and congenital contractures [13] those with a more severe phenotype characterized as Walker-Warburg syndrome often die within the first year of life [14]
	Autosomal recessive muscular dystrophy-dystroglycanopathy type B1 (MDDGB1) (MedGen UID: 461765)	hypotonia at birth joint contractures severe psychomotor retardation inability to walk striking enlargement of the calf and quadriceps muscles absent speech mental retardation enlargement of the cisterna magna and cerebellar hypoplasia [15,16] leg hypertrophy microcephaly grossly delayed motor milestones facial weakness with tendency to keep the mouth open mild macroglossia lower limb stiffness increased serum creatine kinase [17] autistic features diffuse muscle wasting scoliosis cardiomyopathy [18]
	Autosomal recessive muscular dystrophy-dystroglycanopathy type C1 (MDDGC1) (MedGen UID: 332193)	early motor milestones age at onset ranged from 1 to 6 years, with difficulty in walking and climbing stairs slow progression, proximal muscle weakness mild muscle hypertrophy increased serum creatine kinase microcephaly mental retardation (IQ range 50 to 76) [19] shortness of breath easy fatigability left ventricular hypertrophy and dilation of the ventriculars, systolic dysfunction calf and thigh hypertrophy, relative wasting of the scapulohumeral girdle myalgias in the shoulder girdle calf hypertrophy normal cognition [18]
*TRIM32* (Tripartite motif containing 32)	Autosomal recessive Bardet-Biedl syndrome (BBS) (MedGen UID: 395295)	Obesity polydactyly renal anomalies retinopathy hypogonadism learning disabilities [20]
	Limb-girdle muscular dystrophy type 2H (LGMD2H) or Limb-girdle Muscular Dystrophy R8 (LGMDR8) (MedGen UID:78750)	Nonprogressive muscular weakness from infancy [21] slowly progressive dystrophy in the quadriceps and pelvic girdle musculature with facial features waddling gait difficulty rising from the squatting position ’flat smile’ [4] creatine kinase (CK) levels more than 4 times the upper limit of normal. Asymptomatic patients with extremely elevated CK levels (more than 15 times the upper limit of normal) were also considered affected [5] scapular winging moderate hypertrophy of the calves absent deep tendon reflexes exercise-induced myalgia [22] respiratory weakness chronic keratitis paresthesias lost the ability to walk [23]

**Table 3:** Collation of clinical presentation of the Limb-girdle Muscular Dystrophy R8 according to the Human Phenotype Ontology and pathological manifestations in the patient [24].

Term Name	Frequency	Presence in the Patient
Tall stature	Frequent	Not detected
Mask-like facies	Very frequent	Not detected
EMG abnormality	Very frequent	Detected
Increased variability in muscle fiber diameter	Very frequent	Not conducted
Myopathy	Very frequent	Detected
Proximal muscle weakness in lower limbs	Very frequent	Detected
Gait disturbance	Very frequent	Detected
Waddling gait	Very frequent	Detected
Elevated serum creatine kinase	Very frequent	Detected
